# A Conceptual Framework for Integrated Community Care

**DOI:** 10.5334/ijic.5555

**Published:** 2021-02-10

**Authors:** Yacine Thiam, Jean-François Allaire, Paul Morin, Shelley-Rose Hyppolite, Chantal Doré, Hervé Tchala Vignon Zomahoun, Suzanne Garon

**Affiliations:** 1 Institut universitaire de première ligne en santé et services sociaux (IUPLSSS) du Centre intégré universitaire de santé et de services sociaux de l’Estrie – Centre hospitalier universitaire de Sherbrooke (CIUSSSE-CHUS). Hôpital et centre d’hébergement D’Youville, Sherbrooke, QC, Canada; 2 Institut universitaire de première ligne en santé et services sociaux (IUPLSSS) du Centre intégré universitaire de santé et de services sociaux de l’Estrie – Centre hospitalier universitaire de Sherbrooke (CIUSSSE-CHUS), CA; 3 Université de Sherbrooke, CA; 4 Centre intégré universitaire de santé et de services sociaux de la Capitale nationale, CA; 5 Faculté de médecine, Université Laval, CA; 6 Knowledge Translation Component of the Quebec SPOR-SUPPORT Unit, CA

**Keywords:** health care, social care, proximity, community based, temporality, integrated care

## Abstract

**Introduction:**

The various health and social care services provided in a given local area (i.e., place-based) must not only deliver primary care in proximity to the population, but act upstream on the social determinants of health. This type of care, when provided in a holistic and integrated manner, aims to improve the physical and mental health—but also the well-being and social capital—of individuals, families, groups and communities. This type of approach is known as Integrated Community Care (ICC).

**Theory and methods:**

This article was developed from a non-systematic review of scientific and grey literature followed by a qualitative analysis and researcher reflections on ICC.

**Results:**

The article presents the core concepts of ICC, namely temporality, local area, health care, social care, proximity and integration. These concepts are unpacked and a conceptual diagram is set forth to put the dynamic links between the concepts into perspective.

**Discussion and conclusion:**

The purpose of the article is to provide a conceptual clarification of ICC. Three examples of practise (from Switzerland, Quebec [Canada] and Italy) are used as illustrations to provide a better understanding of ICC and to open up horizons.

## Introduction

Health and social care needs differ from one geographic area to another. In socially and materially disadvantaged localities, the complexity and diversity of the problems faced by people and populations constitute a challenge for the health and social care networks. The solutions to these challenges lie in the implementation of local, integrated and community-based health and social care that is anchored in these communities [1].

For a number of years, innovative projects described as integrated community care practises and focusing on local interventions in living environments have been implemented throughout the world [2,3,4]. However, the notions of proximity, local area (place, locality) and integration used to describe this type of intervention are not clearly conceptualised in the literature, despite the recent review of Australian literature on a part of this topic [5] and the TRANSFORM integrated community care initiative [6].

The purpose of this paper is to help conceptually clarify this kind of practise that considers health and social care provided in proximity, taking into account the different temporality of stakeholders and populations in an integrated and place-based manner, which we call integrated community care (ICC) practises. Three concrete examples will be presented to facilitate understanding of the concepts.

## Theory and Methods

This paper is a contribution to updating and reviewing knowledge about the concept of integrated community care. We opted for a narrative-type literature review for the opportunity it offers in the development of new research projects based on the synthesis and interpretation of the results of an unsystematic selection of scientific publications [7,8].

The research and collection of literature were carried out in two steps. At first literature search was conducted around the theme of integrated community care. The keywords related to ICC have been identified in the literature on the subject known by the authors. The following keywords have been targeted: time scale and temporality, territory, place-based, local area, neighbourhood-based as well as health care, social care, proximity and integration. The main selection criteria focused on the theme of local health care or local social care, whether integrated or not, localized or not, and provided by the public health and social care network, the community or the private sector. Subsequently, the literature search in Google Scholar and using snow-ball search strategy focused on the key concepts identified, in order to complement the information collected. These texts are published in scholarly journals or come from the grey literature known to the research team.

We selected 51 papers to undertake the conceptual clarification of the six concepts that constitute ICC. According to our knowledge and analysis of various practises, these concepts are local area and temporality (time and space) as a framework for practices; health care, social care, proximity and integration. A complete reading of these documents was performed to pinpoint definitions of the concepts at the core of ICC. A thematic qualitative analysis of the data was conducted for each concept [9]. The extracted data was processed and systematized using a coding tree (***Appendix 1***) based on four considerations:

1. What approaches (health care, social care or both) were used?
2. Which setting (public network, community setting, private or mixed sector) implemented the intervention?
3. What types of people and populations were targeted?
4. What are the goals or effects/impacts? (Example of the use of the grid in ***Appendix 1***).

## Results

### Local area and temporality

These two concepts are central and give structure to all ICC. People live in a given environment, with its perceived, experienced or conceived features and dynamics. Temporality significantly influences interventions’ success, as citizens and users’ perceptions of time may differ from those of care givers and health and social care systems workers. Temporality also changes according to stage of life or historical evolution of the area.

#### Local area

The local area is a socio-spatial entity, a living environment strongly shaped by its inhabitants, their interpersonal and social dynamics, their demographic characteristics, their history and their culture. It changes physically over the years in terms of the built environment. The local area finds legitimacy in the representations it gives rise to on the symbolic, heritage and imaginary levels [10]. The concept of local area has several dimensions, namely geographic local area, lived local area, perceived local area and designed local area.

1- Geographic local area: As an intervention environment, the geographic area is the service area that allows the health and social care network to ensure continuity of care [11] by taking into account the structure of the territory (public care, community resources, parks, private care and businesses).
2- Lived local area: Refers to the setting of everyday life [12]. It includes the social participation of groups and individuals, who maintain meaningful relationships within the community. The lived local area is shaped from the lived experience of the people inhabiting it [13]. It is thus the place where needs and identities are managed [14]. In the field of health and social care, the lived local area is a strategic place, particularly for public institutions, when it comes to improving efficiency and acting on social and health inequalities [15,16,17].
3- Perceived local area: Refers to the cultural space of social practises, legitimacy and identities. The perceived local area encompasses the geographic identity heritage perceived by each person depending on their life stage. It allows the development of a sense of belonging to the local area according to the personal or collective meaning given to places, past or present, lived or even imagined [18].
4- Finally, the designed local area is the rationalised space of state planning and its management (i.e. municipal districts, borough, etc.) [14].

It should also be noted that the various types of local areas can strongly influence the social determinants of health and health inequalities and thus guide the allocation of certain health expenditures [19]. Knowledge and understanding of the intervention area as a fusion of the geographic local area, the lived local area, the perceived local area and the designed local area is therefore conducive to the provision of ICC [20,21].

#### Temporality

Temporality is “the experience of time and the temporal organization of activities around us. … It is also central to our interactions with each other” [22, p. 33]. In an ICC context, the concept of temporality clarifies the importance of time, both for care givers, who need the flexibility to deliver this type of intervention, and for people, who experience life events at self-pace [23]. Care givers’ relationship to time is linked to the professional realities of their institutions, such as care planning and coordination process [24]. It also relates to the time needed to understand personals’ needs or issues in terms of care. For people, temporality seems to be more strongly shaped by their personal or collective needs and history rather than by the way care services are organised by health and social care structures. As a result, their temporality, whether personal or collective, is as much related to the health and life conditions of each person as it is to the common needs of the community [23]. Moreover, temporality at a personal level also entails a time to assimilate the changes needed to improve their living conditions. Taking into account temporality from a personal perspective also helps better include the multiplicity of personal experiences and relationships to time [25].

The concept of temporality is also cross-cutting, spanning local area, proximity and integration. For instance, temporality can vary from one local area to another in relation to the size of the area, the local services available and the geographic possibilities to access services, but also to the lifestyles, historical and cultural specificities of the populations [1,21]. In terms of proximity (spatial as well as relational), the duration of care provided in the community and the creation of ties with populations vary from one population type to another. As an example, creating ties in a local area with refugees may take a different approach to time related to the culture of those refugees [1,21]. The same observation applies to integration: developing cross-sectorality and interdisciplinarity is a long-term process [26,27]. Care offered in a hospital, ICC care givers or a community partner may have different timeframes. These timeframes need to be discussed when working across sectors or disciplines.

### Health and social care

The health or social care delivered in communities (within a specific local area and temporality emphasis) is usually associated with primary care. Sometimes, however, it will involve specialised care.

#### Health care

“Health is a state of complete physical, mental and social well-being and not merely the absence of disease or infirmity” [28, p. 1].

Health care consists of interventions or treatments involving the use of approaches, technologies or mechanisms for preventive, diagnostic, therapeutic, palliative, rehabilitative or supportive purposes [29]. Three levels of health care can be distinguished: primary care, secondary care and tertiary care.

Primary care is the level of professional care where populations have their first contact with the health system and where the majority of their curative and preventive health needs are met [30]. It focuses on people to better meet their needs. Primary care is based on equity and solidarity and promotes everyone’s right to a better state of health [31]. In the literature, “primary care” and “primary health care” may be used interchangeably. However, as an organisational concept, a distinction needs to be made. Primary health care has a broader and more political connotation [30]. In Canada, for example, and in Quebec in particular, the concepts of primary care and primary health care are sometimes used interchangeably, even though they are philosophically different and do not reflect the same realities of needs and service provision [32]. In Quebec, the health system’s structure translates into first line – second line – third line or general (comprehensive) care – specialized care – ultra-specialized care [33]. In Europe, this structure is divided into primary care, secondary care and tertiary care [34]. Hence, we consider primary care to be aimed at responding to various common health or social problems, provided close to users’ living environment and intended for the general public and special-needs clienteles. Access to primary care is generally direct and the terms for benefiting from it are simple, predictable, clearly defined and known to users [32,35].

While the main role of primary care is to provide care for common health problems, it also plays the secondary role of referring sick people to more specialised care [34]. Secondary health care thus complements primary care and helps treat complex but widespread health problems, while tertiary care is aimed at people with very complex health problems requiring long-term case management. The distinction between secondary and tertiary care comes down to the degree of specialization of care [34,35].

#### Social care

In industrialised countries, social care refers to all care provided by institutions, public or private, to protect and support individuals, families, groups and communities in vulnerable situations (social, economic, physical or cultural). Social care is the set of prevention, rehabilitation and social protection care provided to individuals, families, groups and communities to ensure their well-being and promote their autonomy [36,37]. It is guided by the principles of social justice, human rights, collective social responsibility and respect for diversity [38]. In concrete terms, it includes all public programs, non-profit community care, sometimes private programs, and actions in solidarity with people experiencing difficulties on a temporary or chronic basis. Social care relies on activities based on supportive relationships between care givers (social workers, community organisers, etc.) and people needing care. It also refers to psychosocial practises aimed at social change and development, social cohesion, and the empowerment of persons, families, groups and communities in vulnerable situations [39,40].

When social care concerns the situation of an individual, family, couple or small group, it requires a psychosocial assessment. However, when it pertains to a community or group, it instead demands an ecological approach. Social care activities in community settings involve multidimensional actions including mobilisation, awareness, representation and coordination tasks, among others, in order to initiate changes in individuals and their environment as well as in the political and social structures affecting the targeted populations [41].

In Quebec, social care (named “social services”) is sometimes supported by community organisers whose mission is to foster stronger local communities through the creation of support, mutual aid and solidarity networks [42]. While elsewhere in the world, leadership in community development is often assumed by the community itself and non-profits [43], in Quebec, this action to support intervention is equally divided between non-profits and the public network.

### Proximity

Applied to the field of health and social care, proximity refers to care provided as close to populations as possible, in a familiar environment (housing, medical clinics, local businesses, neighbourhood premises, community organisations, other partners in a local area, parks, etc.), in order to keep people at the lowest possible intensity of level of care so that the greatest portion of their health and social care needs are easily addressed, diagnosed and treated in primary care [19]. Our concept of proximity has both a spatial and a relational dimension.

Spatial proximity entails the visibility of care givers and infrastructures in the environment and presupposes “physical proximity” [44]. Spatial proximity in service delivery prompts stakeholders and care givers to be proactive and to “reach out” to populations, particularly those that are marginalised, vulnerable or in need of assistance and support, in order to reduce the gap separating them from the social and cultural norm [44]. It also refers to the visibility, accessibility and availability of care givers and treatment places [21,45]. Moreover, it can be named in various ways, such as place-based, community-based, neighbourhood-based, etc. [21,45].

While spatial proximity implies an understanding of health and social care based on spatial realities [46], the relational dimension is also important. Indeed, the relational dimension requires health and social care providers to be flexible and adaptable, and to strive to adapt service offerings to the specific needs of populations, in order to foster professional collaboration [47]. It is based on the quality of the relationship, such as respect, openness, understanding and compassion, which helps create and maintain bonds of trust with people [21]. This relational proximity is also linked to the people-centred care partnership to be set up with service users, citizens and communities as well as health literacy aimed at empowering individuals, families and communities [48]. As a result, relational proximity allows interaction with citizens and communities as co-producers of care, health improvement and social capital enhancement within the community [49], in line with the October 2018 *Astana Declaration on Primary Health Care* [50]. This approach builds on the strengths of individuals and the community, aiming to empower people. Thus, spatial and relational proximity is conceived from a global perspective of the development of individual and community well-being.

### Integration

In 2016, WHO Europe mentioned the lack of an unambiguous conceptual definition for the concept of integration in the field of health and social care due to its polymorphic nature. Indeed, the realities and perspectives that underlie this concept are likely to be shaped as much by the views and expectations of different health systems as by their actors [51]. In Quebec, integrated care is defined as care whose delivery processes are organised in such a way as to form a coherent whole from the point of view, generally, of the people for whom they are intended [52]. Thus, integration can be defined as connectivity between health and social care [53] with the aim of improving clinical outcomes in terms of effectiveness and efficiency, but also user satisfaction. Our concept of integration entails action to correct gaps in the services path of users receiving simultaneous but separate health and social care. The integration of the two types of care (health and social care) presupposes collaboration and a shared vision of the roles and responsibilities of the players intervening on the same territory. It also aims to provide a better response to the needs of users and their families [35,54]. This concept of integration involves two dimensions: interdisciplinary and cross-sectoriality.

Interdisciplinarity, in a context of integrated care, involves the collaborative work of health and social workers to obtain a global, common and unified understanding of users’ needs, thus enabling concerted action and a complementary sharing of tasks. [26,55]. Sometimes used indiscriminately with the concept of multidisciplinarity, interdisciplinarity goes beyond the mere grouping of workers from several disciplines (medicine, nursing, social care, etc.). It demands concerted and coordinated action.

Cross-sectoriality, in a context of integration of health and social care, is an important dimension that refers to the mobilisation of stakeholder’s from more than one intervention sector engaged in complementary action. The aim is to rely on each stakeholder skills and resources to meet certain needs clearly recognised in the community [27,56]. This approach requires adaptive management of complexity, as health and social care players are no longer the sole decision-makers [57]. In the *Adelaide Statement on Health in All Policies*, cross-sectoriality is stated to be an important element in promoting populations’ health and well-being:

To harness health and well-being, governments need institutionalized processes which value cross-sector problem solving and address power imbalances. This includes providing the leadership, mandate, incentives, budgetary commitment and sustainable mechanisms that support government agencies to work collaboratively on integrated solutions [58, p. 2].

### Targeted outcomes

The targeted outcomes of these practises relate not only to improved performance of the health or social care system, but also to improved health and well-being of individuals and communities [47]. Thus, these practises can impact social capital in the community, social networks, social cohesion, and participation in co-production [59]. Finally, another sought-after outcome is health equity, as these practises are provided in disadvantaged communities at various levels, but nevertheless with assets that can constitute intervention leverage [57].

**Figure 1:**
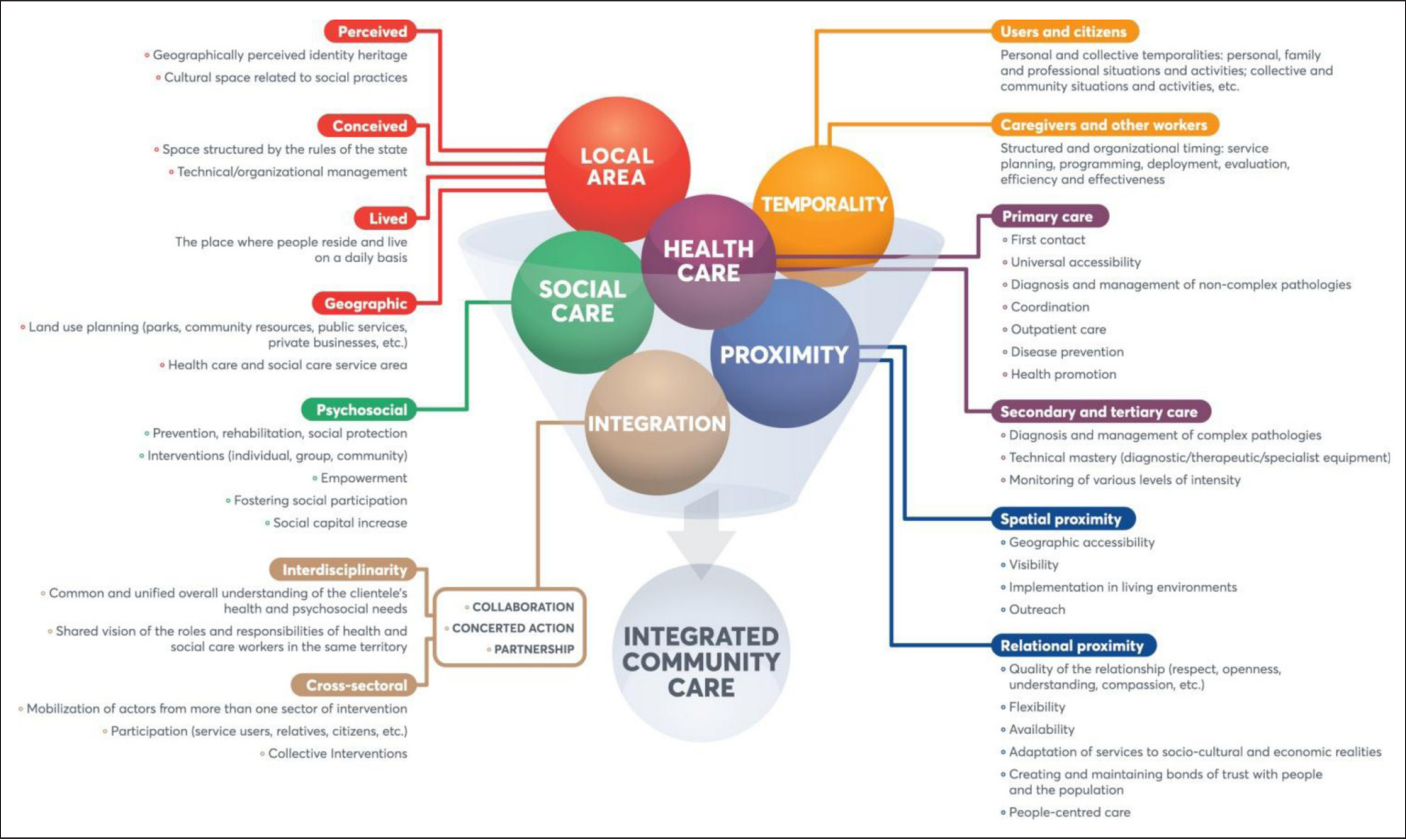
Conceptualisation of integrated community care.

The following diagram (***Figure 1***) sums up the concepts and their components as presented above.

Based on this conceptual framework, we define integrated community care (ICC) as an interweaving of localized and temporalized health and social care interventions provided in proximity (spatial and relational) in an interdisciplinary and cross-sectoral manner. ICC aims to improve physical and mental health, well-being and empowerment, as well as to facilitate access to and use of care, particularly among disadvantaged populations or those not served by the health and social care system.

## Discussion: concrete examples

In order to provide concrete examples, four parameters were taken into account in the analysis of the selected texts: 1) the environment in which the intervention was carried out; 2) the targeted population; 3) the objectives pursued; and 4) the approach used.

Several sectors (public network, community sector and private sector) carry out various interventions with populations, particularly those living in precarious situations. The main objectives of these interventions are to act on the determinants of health and well-being; to improve the social capital of the territory and its population; to reduce social inequalities in health; to promote access to and use of services; and to improve the physical and mental health of the targeted individuals and populations. Given that these types of interventions generally have the same targets and objectives and can be implemented in various settings, whether in proximity or not, localized or not, we have classified them according to the approaches used. Along these lines, we have identified three types of interventions related to ICC: integrated community health care, integrated community social care and integrated community care.

The illustrations below are real cases from various places around the world. They are all delivered in close proximity and adapted to the spatial and temporal specificity of the local area served and the targeted populations. The last case presented is the one integrating all the components of ICC. However, various approaches largely integrate the components of ICC, focusing mainly on health care or on social care. We illustrate each case with a figure adapted from ***Figure 1***.

### Type 1 “Integrated community health care”: Consultation ambulatoire mobile de soins communautaires (CAMSCO) [mobile outpatient community-care consultation] (Switzerland) [60,61]

**Figure 2:**
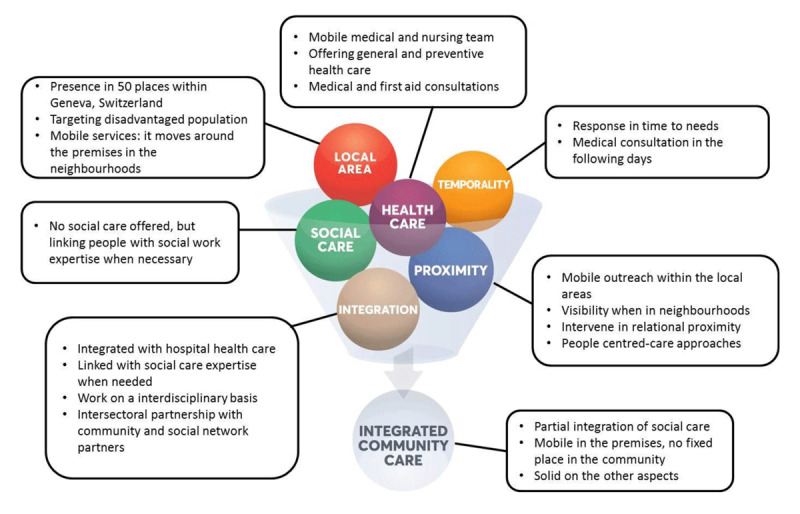
ICC concepts in action – the Geneva CAMSCO mobile outpatient community-care consultation case.

1. Setting: A hospital (Hopitaux universitaires de Genève) offering a service specialised in helping the most disadvantaged, whose mission is to fulfil community health care needs in Geneva. The team has a mobile presence in 50 places on the area of the canton. The initiative was created in 1997 following the enactment of the *Federal Law on Health Insurance*.
2. Targeted population: Anyone over the age of 16 in a precarious situation (homelessness; illegal immigration; social, family or professional isolation), in particular those without a family doctor or health insurance.
3. Objectives pursued: To promote access to health care for people in vulnerable situations and to link up if needed with social care structures and other health care services within the hospital.
4. Approach used: CAMSCO operates on an interdisciplinary basis, combining medical and nursing care and linking people with social work expertise when necessary. The cross-sectoral partnership with community and social network partners contributes to addressing vital needs related to the social determinants of health. In concrete terms, CAMSCO is a mobile structure with a medical team, offering general and preventive health care in relational and spatial proximity. It offers medical and first aid consultations that are easily accessible within the following days. It moves around the premises in the neighbourhoods or among the population, and embraces a people-centred care approach. ***Figure 2*** presents a summary of the components put into action by CAMSCO, according to each key ICC concept.

### Type 2 “Integrated community social care”: Intervention de quartier [neighbourhood intervention] in Sherbrooke (Quebec, Canada) [1,4]

**Figure 3:**
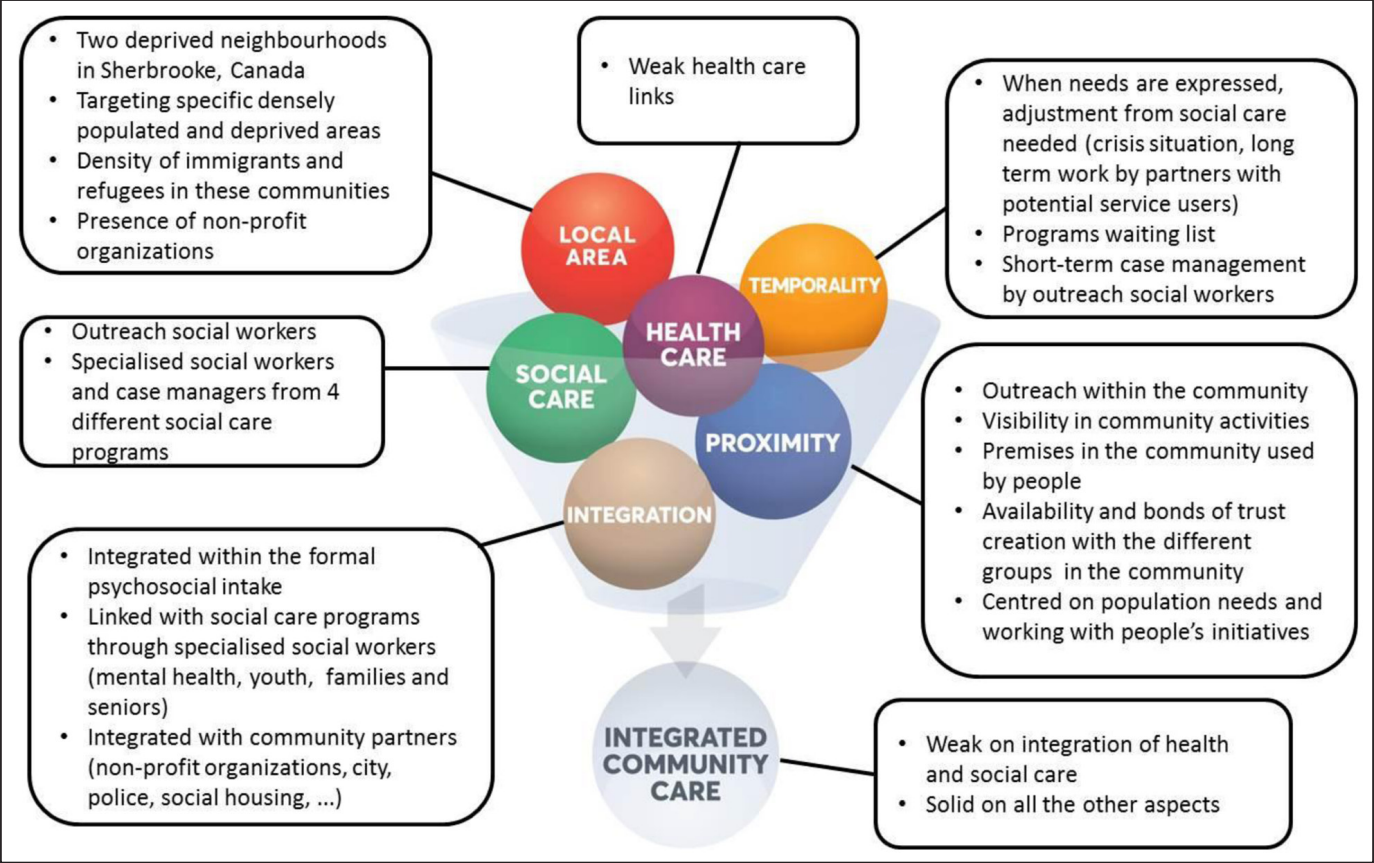
ICC concepts in action – Sherbrooke neighbourhood intervention case.

1. Setting: Centre intégré universitaire de santé et de services sociaux de l’Estrie – Centre hospitalier universitaire de Sherbrooke (CIUSSSE-CHUS) in partnership with the community. The social care services are deployed in two deprived neighbourhoods in the city of Sherbrooke. This social care intervention began in 2009.
2. Targeted population: populations residing in two intervention areas (densely populated neighbourhoods) of the city of Sherbrooke, living in precarious situations.
3. Objectives pursued: To improve the conditions and quality of life of citizens from these two deprived neighbourhoods by implementing a flexible, proactive and adapted social care intervention practise. This is accomplished by taking action on the social determinants of health with a view to social problem prevention, health promotion and community development. This intervention practise also aims to optimise work in partnership with all the stakeholders across the local areas.
4. Approach used: Psychosocial approach based on taking into account the particularities of individuals, their networks and local areas; action in spatial and relational proximity; proactivity, outreach, flexibility and adaptability of interventions. Temporality is taken into consideration, especially when working with immigrants or people waiting for specialised care. ***Figure 3*** presents a summary of the elements implemented by the Sherbrooke neighbourhood intervention, according to each key ICC concept.

### Type 3 “Integrated community care”: The community that promotes health. Trieste’s micro-territories for equity (Italy, Europe) [62,63]

**Figure 4:**
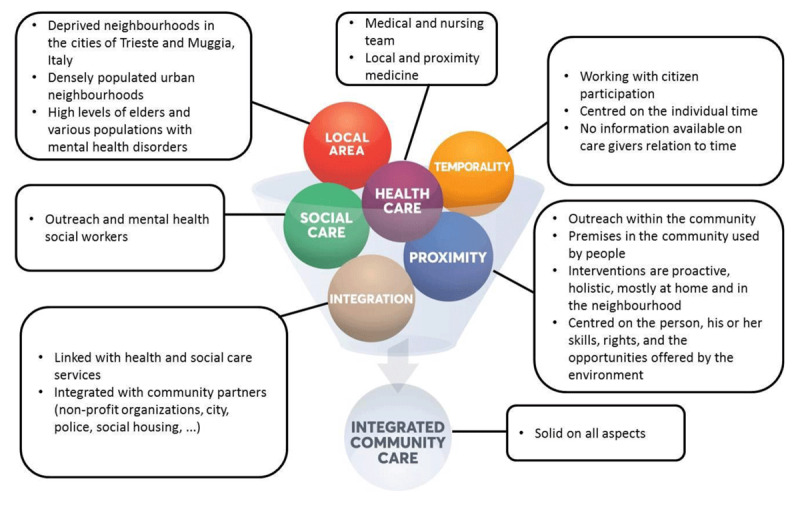
ICC concepts in action – Trieste’s micro-territories for equity case.

1. Setting: Integrated Health Agency of Trieste, the Municipalities of Trieste and Muggia and the Public Housing Office of Trieste. The health and social care services are provided in deprived neighbourhoods in the municipalities of Trieste and Muggia. This intervention was initiated in 2004.
2. Targeted population: Populations of disadvantaged and densely populated urban neighbourhoods. These neighbourhoods have high levels of elders and various populations with mental health disorders.
3. Objectives pursued: To improve living conditions by developing interventions on health promotion and social disease prevention, fostering access to health and social care, and increasing social capital.
4. Approaches used: Approach based on extensive experience with localized mental health care and cross-sector partnership (“Habitat” project, started in 1998). Interventions are thus carried out on the basis of a culture of local and proximity medicine and social care centred on the individual, his or her skills, rights, and the opportunities offered by the environment. The approach is conducive to activating and deploying an alliance with the community in order to develop social capital through political-strategic action toward democratically expanding citizen participation. Interventions are proactive, holistic, mostly at home and in the neighbourhood, and in relation with the resources present in the area in order to avoid hospitalisation. ***Figure 4*** presents a summary of the elements implemented by Trieste’s micro-territories intervention, according to each key concept of the ICC.

### Case studies analysis

The three case studies presented provide a glimpse of the variety of ICC practices. The first case is mainly composed of health care. The second is centred on social care. The last one is an advanced integration of health and social care. All of them share the following common features: cross-sectoral approaches in connection with partners in the community, people-centred care approaches, relational proximity, outreach and visibility in the community, targeting a specific local area. With regard to temporality, the information held sometimes lacked precision, but the case studies seem to take the concept into account differently. The CAMSCO case reaches populations by being mobile and offering them medical consultations adapted to their needs in the following days. The case of Sherbrooke neighbourhood intervention uses its social outreach workers to compensate for waiting lists in specialised services and thus avoid losing the established bond of trust. The case of Trieste and Muggia involves intervention at home as well as in a neighbourhood premises, making it possible to be proactive and to harmonise the temporality experienced by the individuals or by the care givers.

## Conclusion

The conceptualization process presented in this paper has helped to clarify the concepts that make up ICC. These interventions combine health care and social care activities provided in a specific spatio-temporal context, in spatial and relational proximity, integrated and centred on the needs of the inhabitants of a territory. The definition of ICC can serve as a basis for documenting and evaluating existing practises and supporting reflection on their development and the implementation of innovations internationally. In light of the TRANSFORM international initiative [6], this conceptualization process will, we hope, provide insight into these approaches.

## Appendix

**Appendix 1:** Use of the coding tree as an example for the neighbourhood intervention in Sherbrooke.

INTERVENTION	NEIGHBOURHOOD INTERVENTION IN SHERBROOKE (QUEBEC, CANADA)

**Sources**	– Morin, P., Benoît, M., Dallaire, N., Doré, C. & LeBlanc, J. (2013). L’intervention de quartier à Sherbrooke, ou quand le CLSC s’installe à la porte d’à côté. Nouvelles pratiques sociales, 26, (1), 102–117. *https://doi.org/10.7202/1024982ar* – *http://www.csss-iugs.ca/c3s/data/files/12_01_Cahier_recherche_Paul_Morin.pdf* – *http://www.csss-iugs.ca/c3s/data/files/IUPL/Rapp_Rech_Eval_Deploiement_Interv_Chang_Eq_Quartier.pdf*

**Intervention type**	Psychosocial

**Intervention Objectives**	Improving the conditions and quality of life of citizens from two disadvantaged neighbourhoods in Sherbrooke

**Targeted population**	Populations residing in these two neighbourhoods

**Targeted social determinants of health**	Social support, access to housing, food security, social participation

**Targeted health problems**	No information

**Activities**	Proactive psychosocial intake; Resident networking; Mobilisation and support of informal resources; Liaison and clinical support with community partners; Advisory resource; Reaching out

**Partners implementing the intervention**	Centre de sante et de services sociaux – Institut universitaire de geriatrie de Sherbrooke (CSSS-IUGS), now named Centre intégré universitaire de santé et de services sociaux de l’Estrie – Centre hospitalier universitaire de Sherbrooke (CIUSSSE-CHUS) (leader), close partnership with community groups
